# Supplementary material for: Dendrimers with Tetraphenylmethane Moiety as a Central Core: Synthesis, a Pore Study and the Adsorption of Volatile Organic Compounds
Source: Int J Mol Sci. 2022 Sep 22;23(19):11155. doi: 10.3390/ijms231911155 (PMC9570496; doi:10.3390/ijms231911155)
Supplement: Supplementary file 1 [file ijms-23-11155-s001.zip › ijms-1898744-supplementary-update.pdf]

International Journal of *Molecular Sciences*

Support Information

Dendrimers with Tetraphenylmethane Moiety as a Central Core: Synthesis, a Pore Study and the Adsorption of Volatile Organic Compounds

Zi-Ting Gu [1], Chung-Hao Tzeng [1], Hung-Jui Chien [2], Chun-Chi Chen [2] and Long-Li Lai [1,*]

[1] Department of Applied Chemistry, National Chi Nan University, No. 1 University Rd., Puli, Nantou 54561, Taiwan
[2] Department of Environmental Engineering and Science, Feng Chia University, Taichung 40724, Taiwan
* Correspondence: lilai@ncnu.edu.tw; Tel.: +886-49-2910960 (ext. 4976)

Figure S1. The mass spectrum of dendrimer **TAPM-4Den**……………………………………………………..………1

Figure S2. Thermogravimetric analysis of dendrimers **TAPM-4Den** from 50 to 850 °C at a heating rate of 10 °C min^{-1} under an N_2 atmosphere……………………………………………………………………………………..2

Figure S3. The CO_2 sorption isotherms of dendrimers (a) **TAPM-8Den** and (b)**TAPM-4Den** at 273 and 298 K……..…2

Figure S4. The FT-IR spectrum of dendrimers **TAPM-8Den** and **TAPM-4Den**..3

Figure S5. The ^{1}H-NMR spectra of **TAPM-8Den** and **TAPM-4Den** after adsorbing VOCs..…..4

Figure S6. The ^{1}H-NMR and ^{13}C-NMR spectra of dendrimers **TAPM-8Den** and **TAPM-4Den**...…………..….…………..11

Section S1. Estimation of isosteric heats of gas adsorption…………………………………………………………………..13

Figure S1. The mass spectrum of dendrimer **TAPM-4Den**.

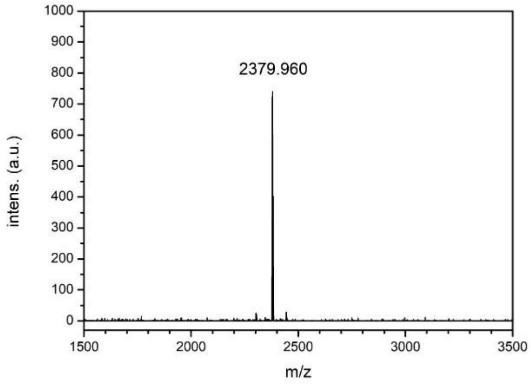

Figure S2. Thermogravimetric analysis of dendrimers **TAPM-4Den** from 50 to 850 °C at a heating rate of 10 °C min^{-1} under an N_2 atmosphere.

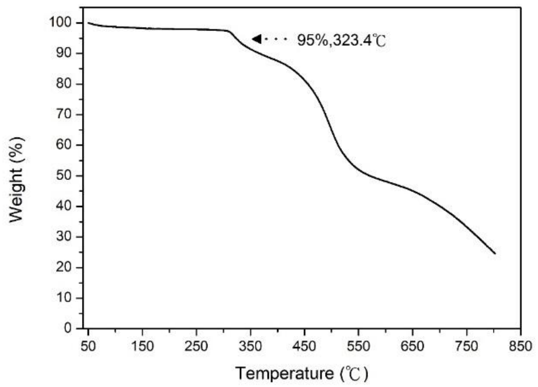

Figure S3. The CO_2 sorption isotherms of dendrimers (**a**) **TAPM-8Den** and (**b**)**TAPM-4Den** at 273 and 298 K.

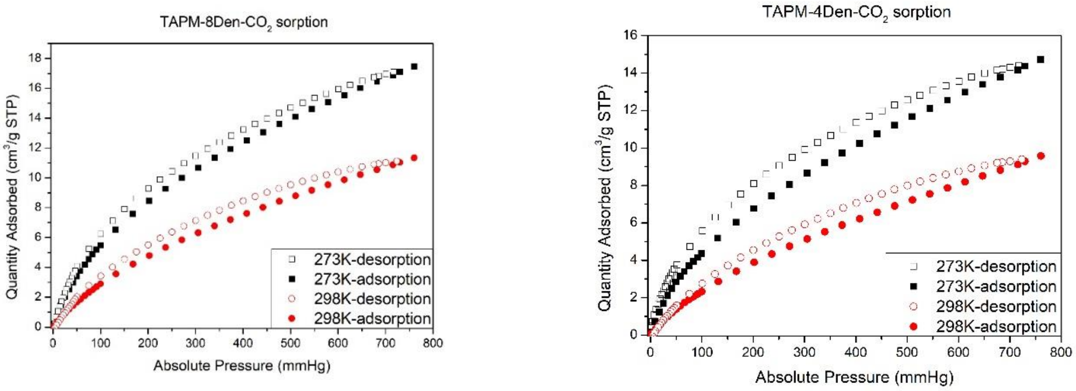

(**a**) (**b**)

Figure S4. The FT-IR spectrum of dendrimers **TAPM-8Den** and **TAPM-4Den.**

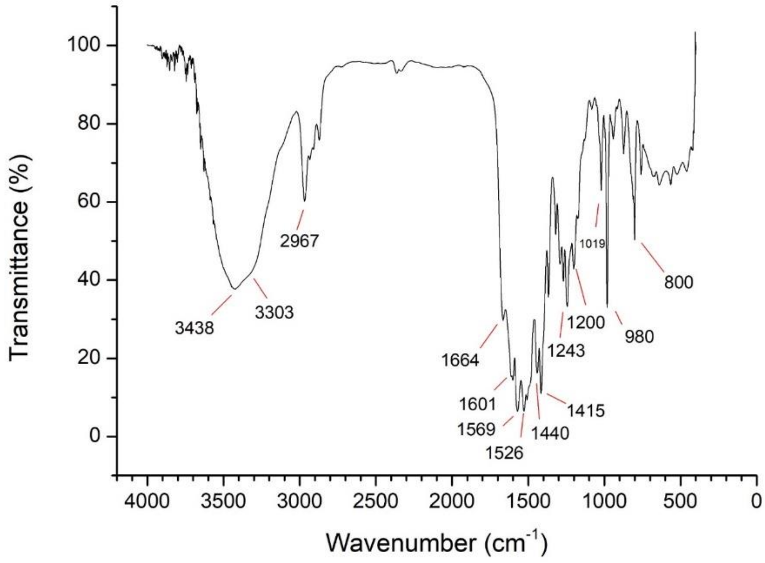

FT-IR spectrum of **TAPM-8Den**

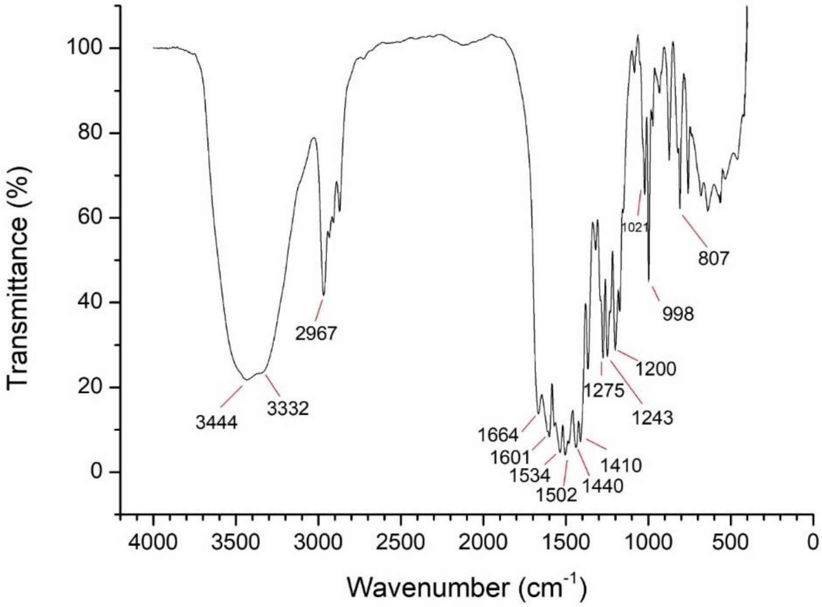

FT-IR spectrum of **TAPM-4Den**

Figure S5. The ^{1}H-NMR spectra of **TAPM-8Den** and **TAPM-4Den** after adsorbing VOCs; the red dots indicate the chemical shifts for calculating the adsorbed ratio.

(A) adsorbing hexane:

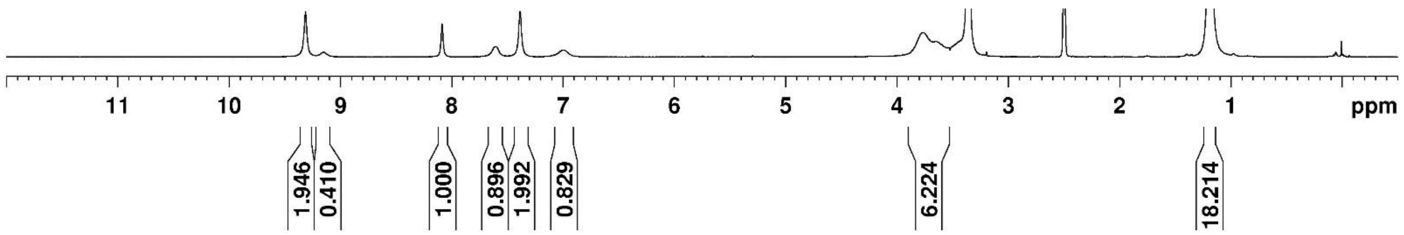

^{1}H-NMR of **TAPM-8Den** DMSO-D$_6$

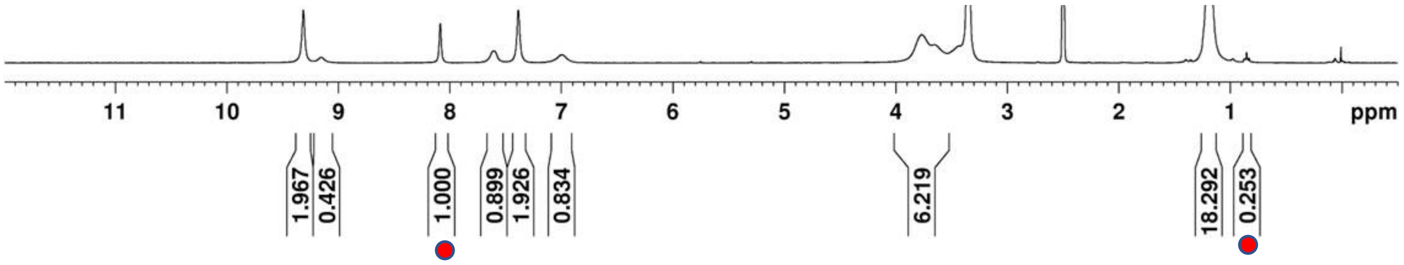

^{1}H-NMR of **TAPM-8Den** after adsorbing hexane in DMSO-D$_6$

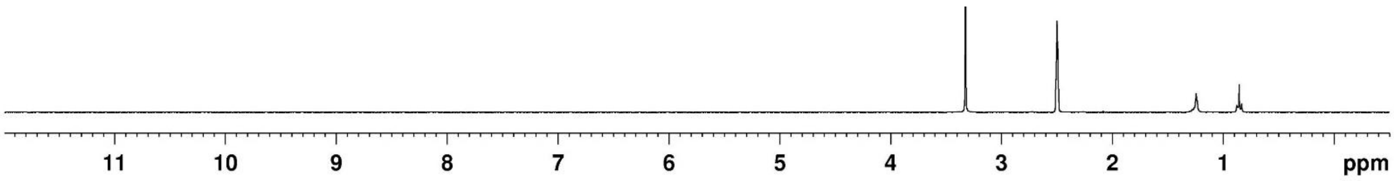

^{1}H-NMR of hexane in DMSO-D$_6$

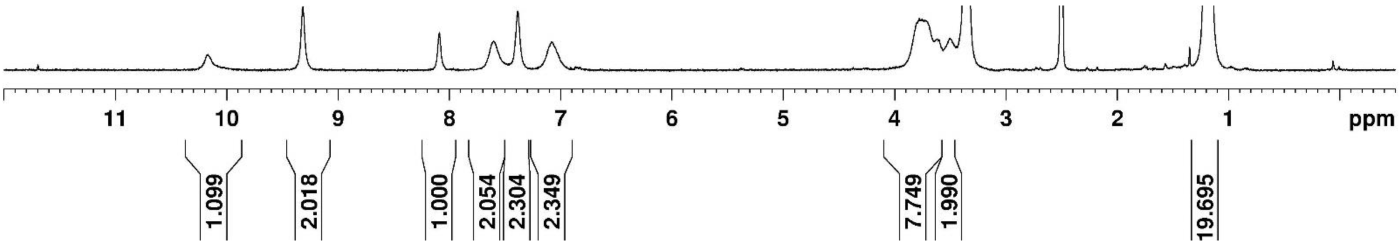

^{1}H-NMR of **TAPM-4Den** in DMSO-D$_6$

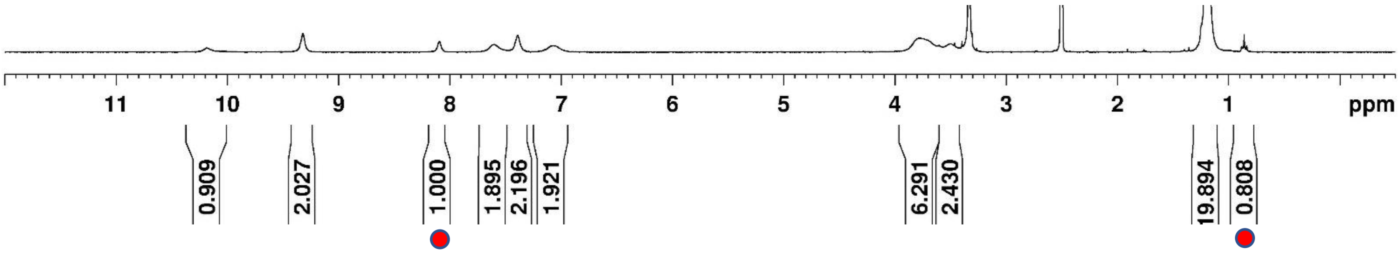

^{1}H-NMR of **TAPM-4Den** after adsorbing hexane in DMSO-D$_6$

(B) adsorbing benzonitrile:

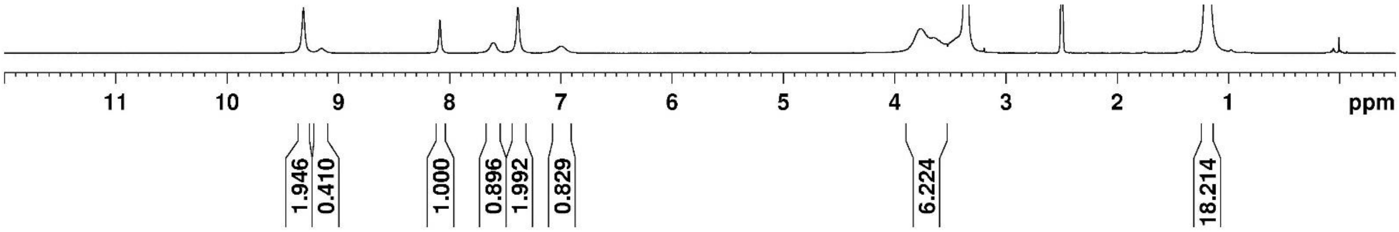

^{1}H-NMR of **TAPM-8Den** DMSO-D$_6$

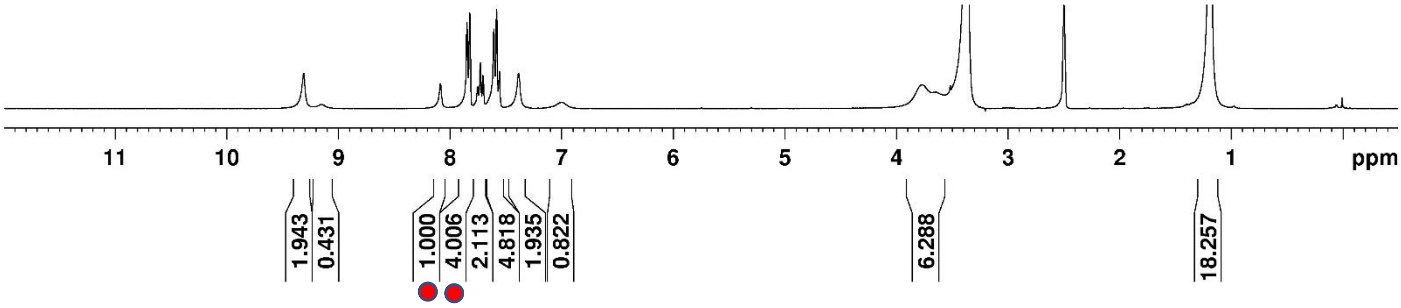

^{1}H-NMR of **TAPM-8Den** after adsorbing benzonitrile in DMSO-D$_6$

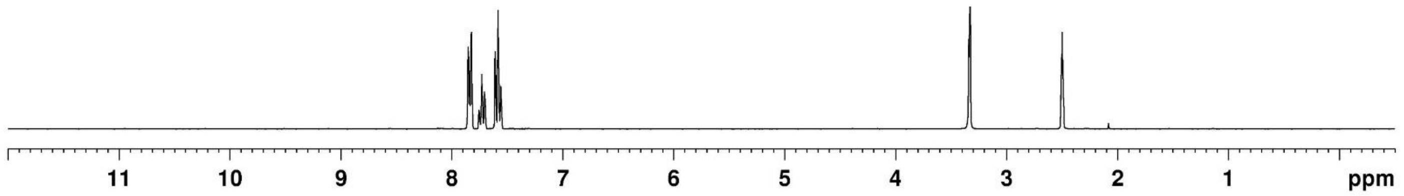

^{1}H-NMR of benzonitrile in DMSO-D6

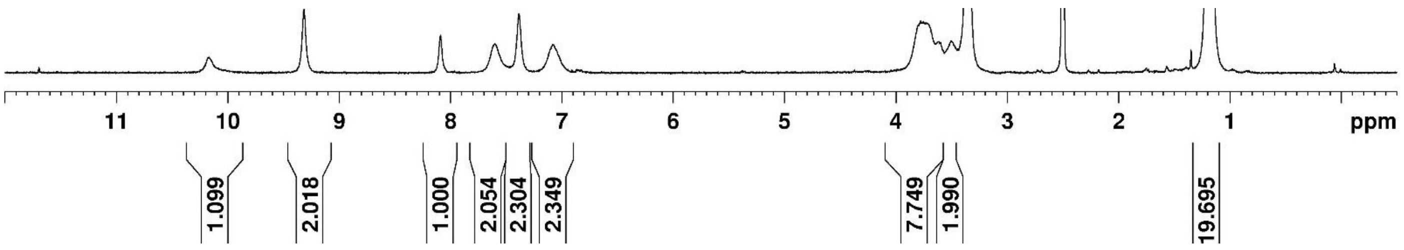

^{1}H-NMR of **TAPM-4Den** in DMSO-D6

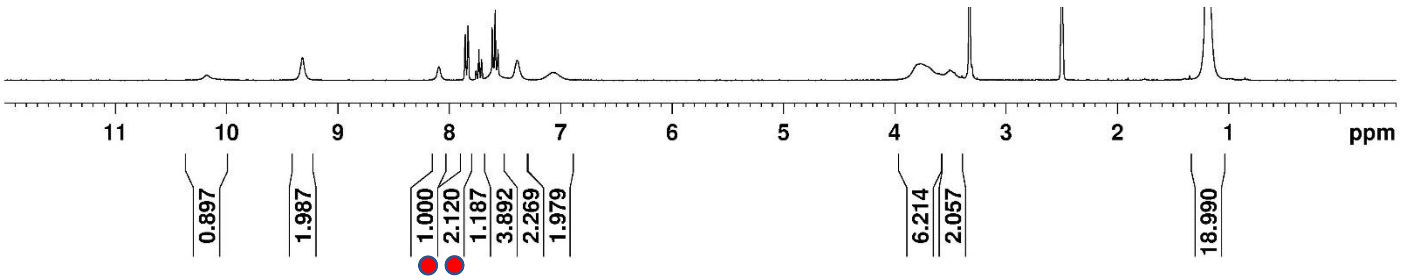

^{1}H-NMR of **TAPM-4Den** after adsorbing benzonitrile in DMSO-D6

(C) adsorbing nitrobenzene:

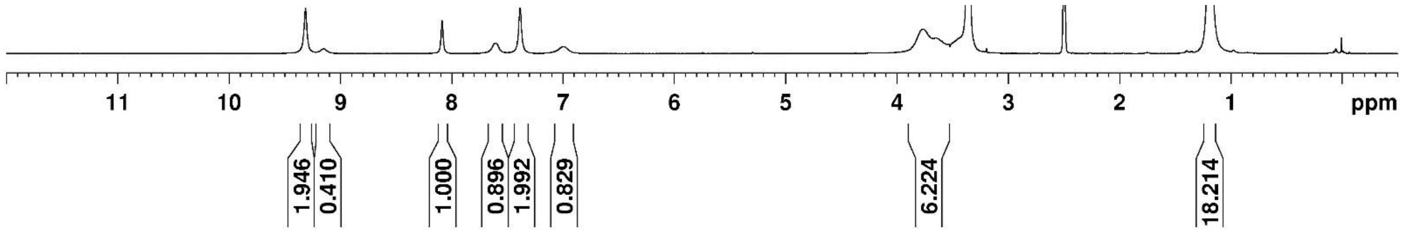

^{1}H-NMR of **TAPM-8Den** DMSO-D6

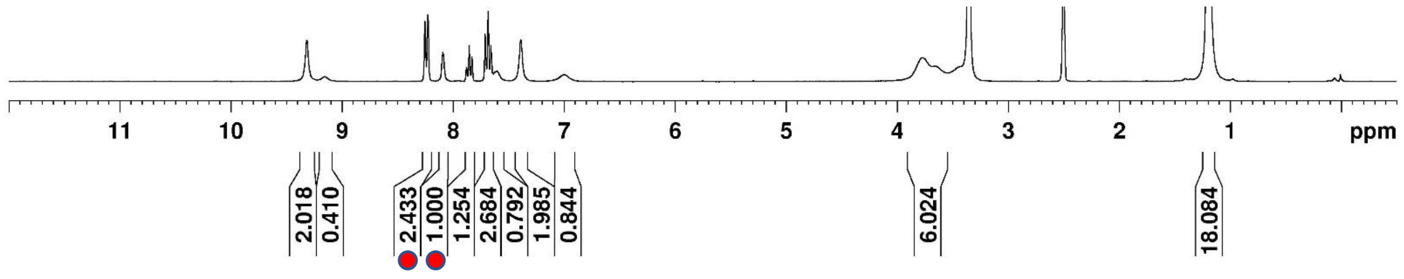

^{1}H-NMR of **TAPM-8Den** after adsorbing nitrobenzene in DMSO-D6

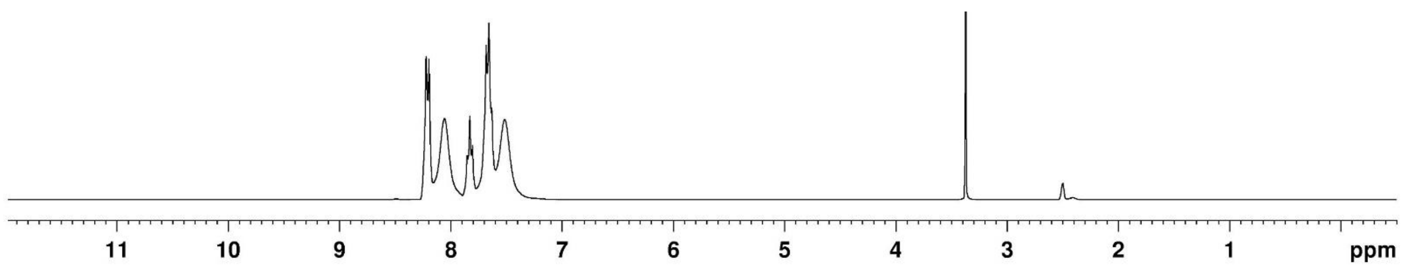

^{1}H-NMR of nitrobenzene in DMSO-D$_6$

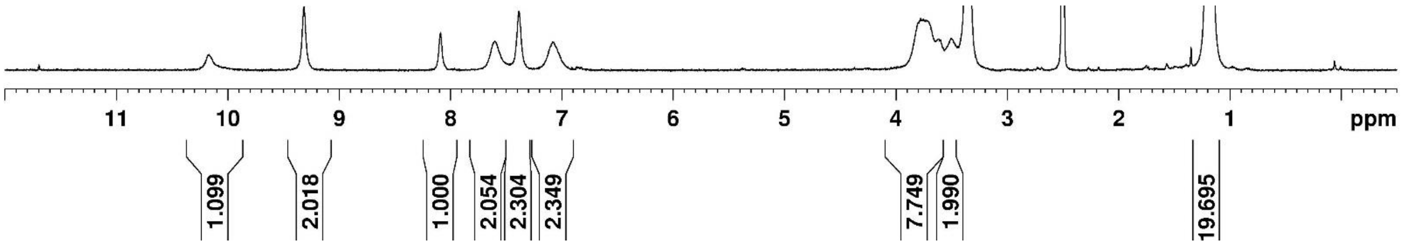

^{1}H-NMR of **TAPM-4Den** in DMSO-D$_6$

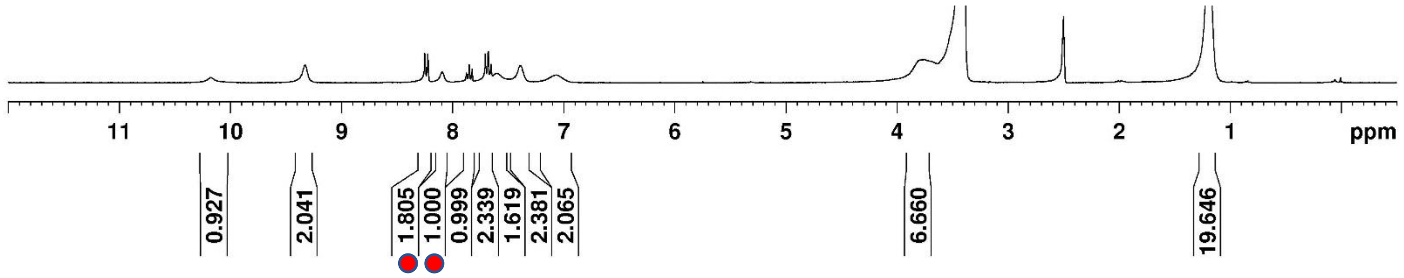

^{1}H-NMR of **TAPM-4Den** after adsorbing nitrobenzene in DMSO-D$_6$

(D) adsorbing toluene:

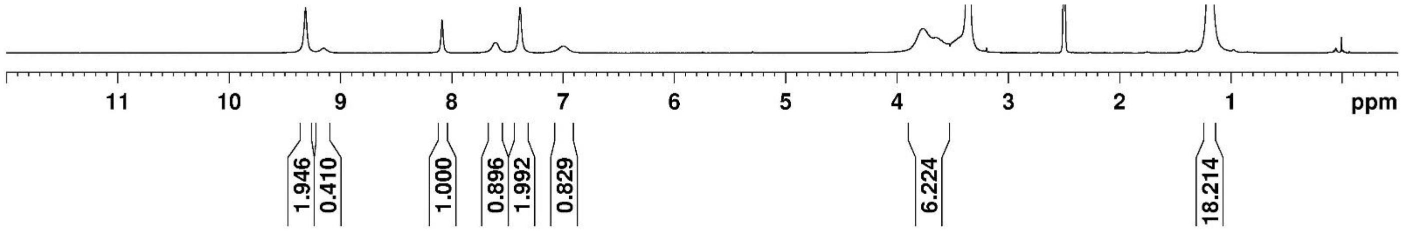

^{1}H-NMR of **TAPM-8Den** DMSO-D$_6$

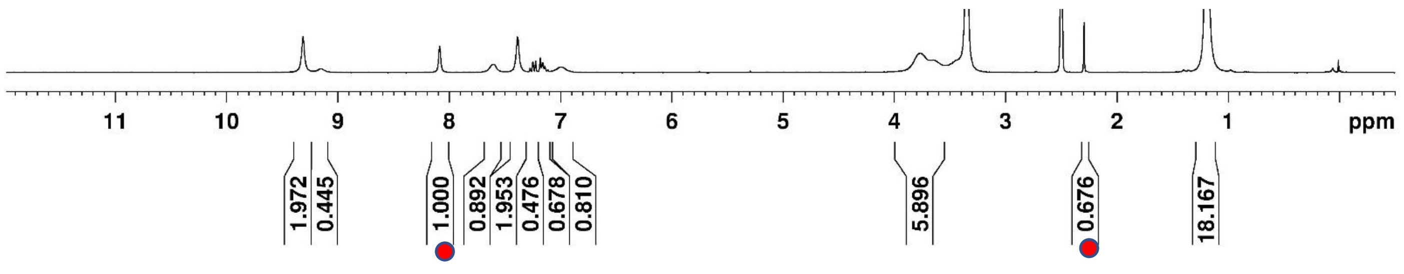

^{1}H-NMR of **TAPM-8Den** after adsorbing toluene in DMSO-D$_6$

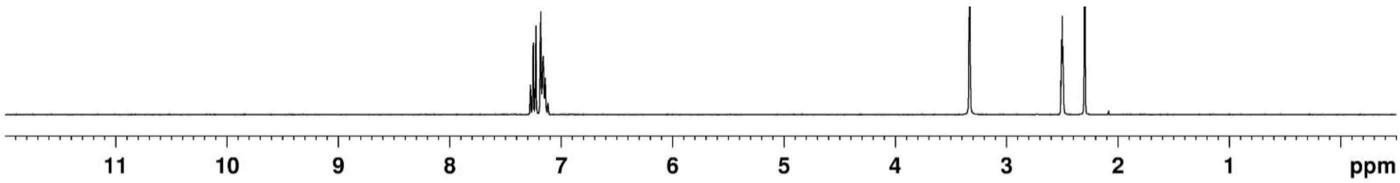

^{1}H-NMR of toluene in DMSO-D$_6$

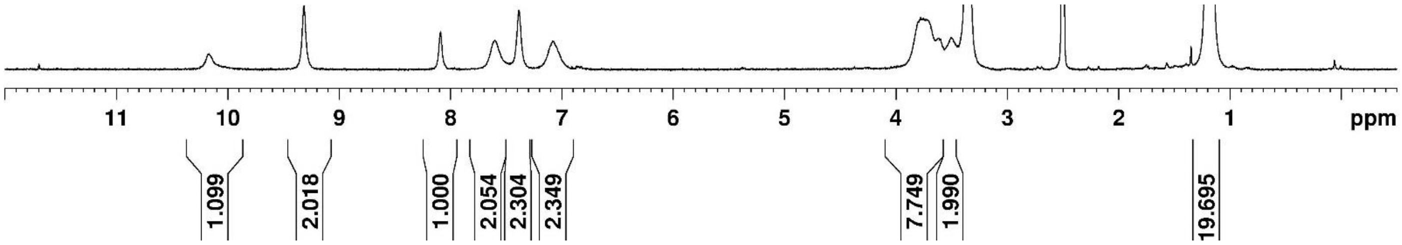

^{1}H-NMR of **TAPM-4Den** in DMSO-D$_6$

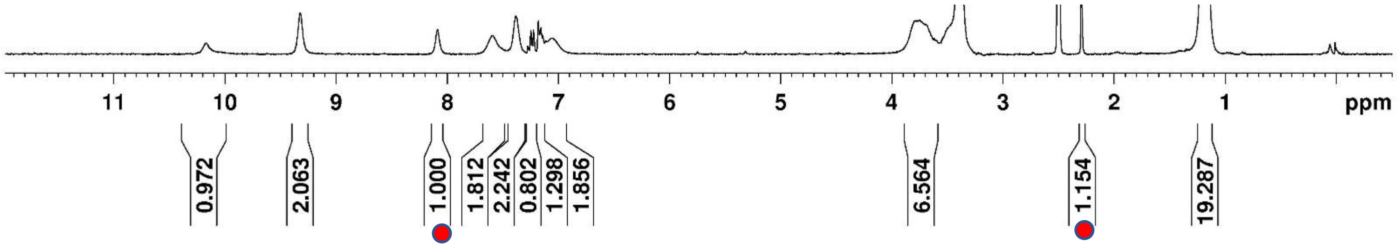

^{1}H-NMR of **TAPM-4Den** after adsorbing toluene in DMSO-D$_6$

(E) adsorbing *o*-xylene:

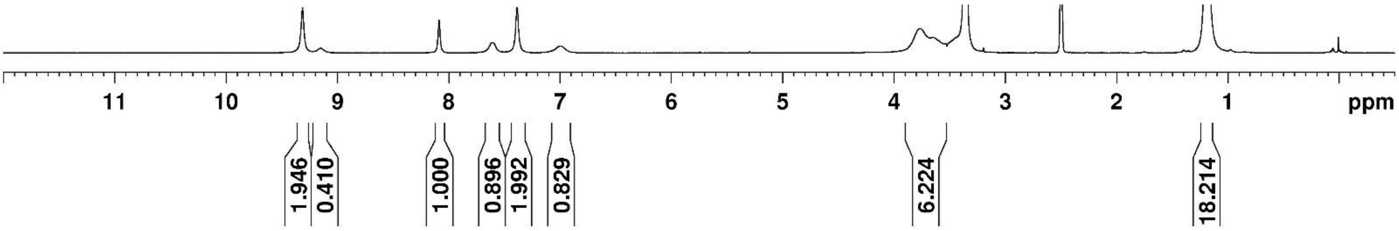

^{1}H-NMR of **TAPM-8Den** DMSO-D$_6$

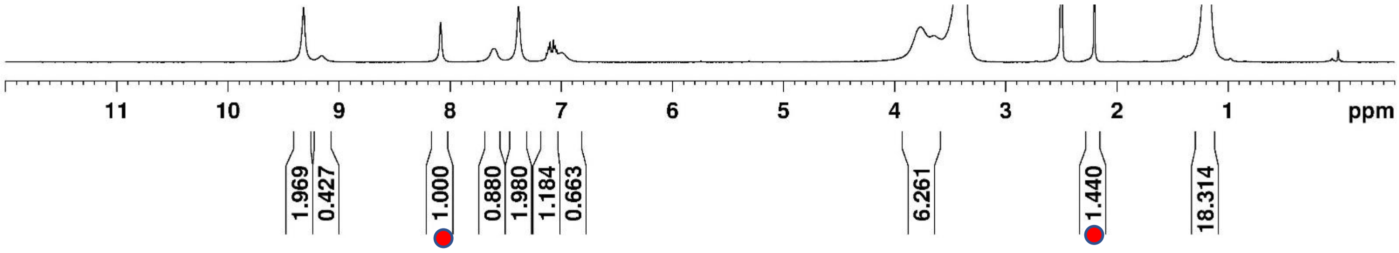

^{1}H-NMR of **TAPM-8Den** after adsorbing *o*-xylene in DMSO-D$_6$

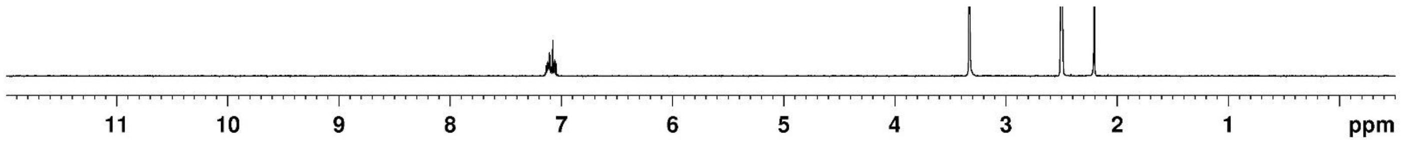

^{1}H-NMR of *o*-xylene in DMSO-D$_6$

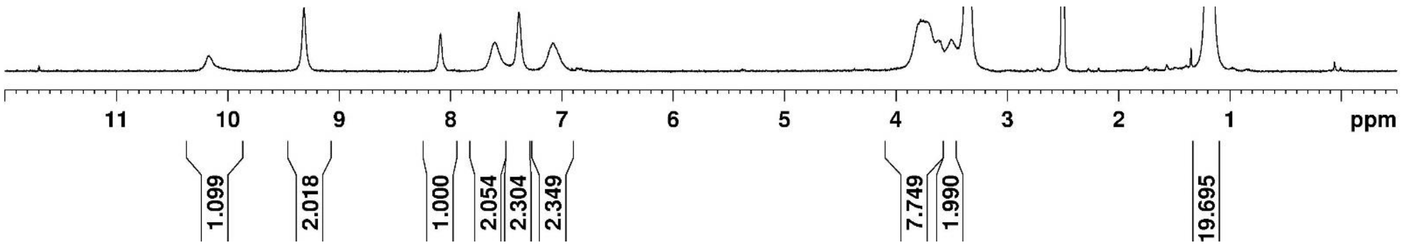

^{1}H-NMR of **TAPM-4Den** in DMSO-D$_6$

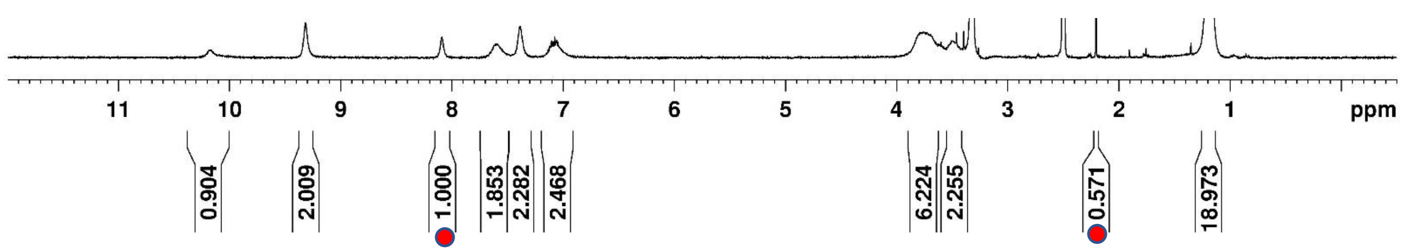

^{1}H-NMR of **TAPM-4Den** after adsorbing *o*-xylene in DMSO-D$_6$

(F) adsorbing *m*-xylene:

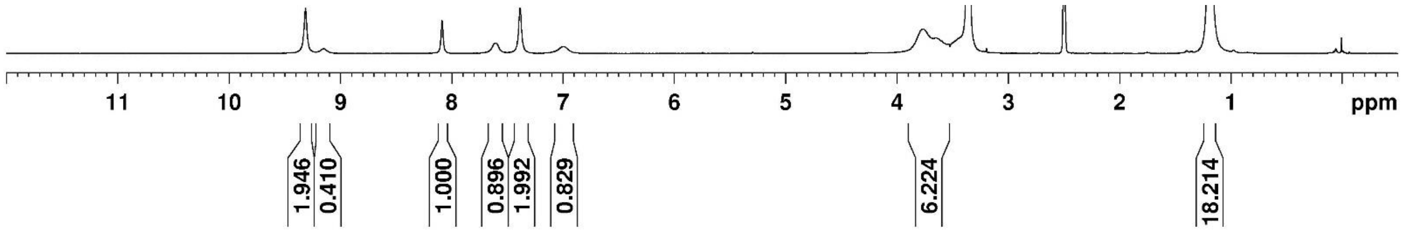

^{1}H-NMR of **TAPM-8Den** DMSO-D$_6$

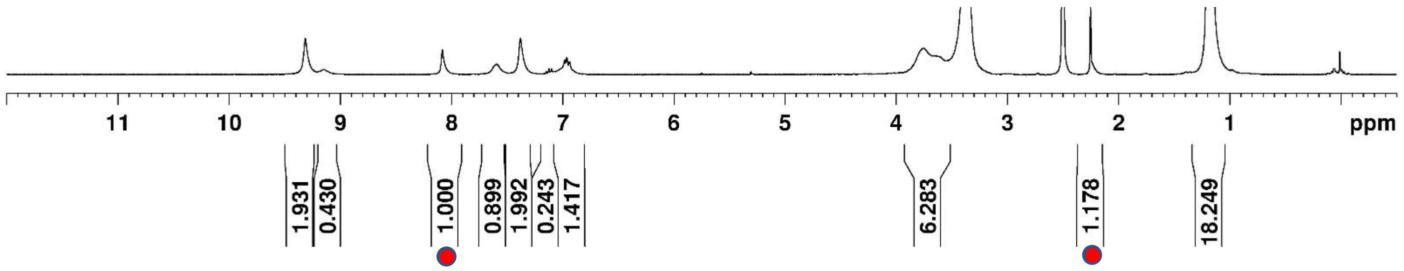

^{1}H-NMR of **TAPM-8Den** after adsorbing *m*-xylene in DMSO-D$_6$

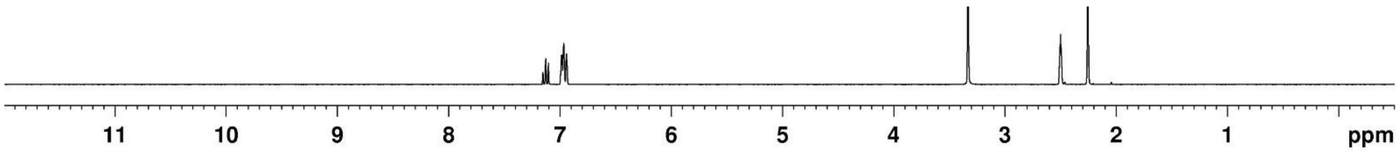

^{1}H-NMR of *m*-xylene in DMSO-D$_6$

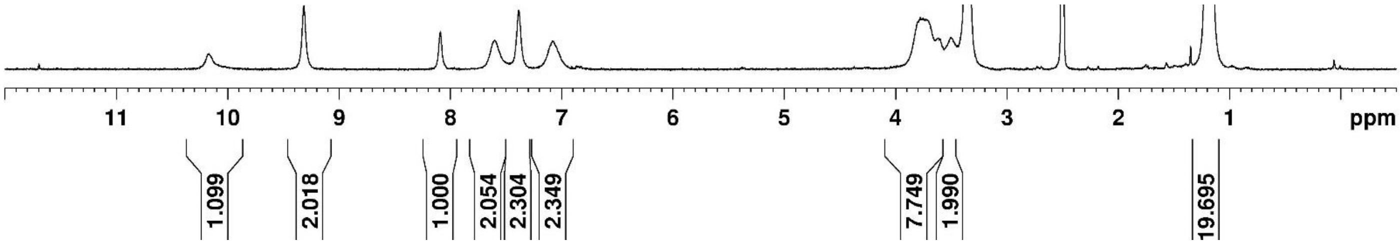

^{1}H-NMR of **TAPM-4Den** in DMSO-D$_6$

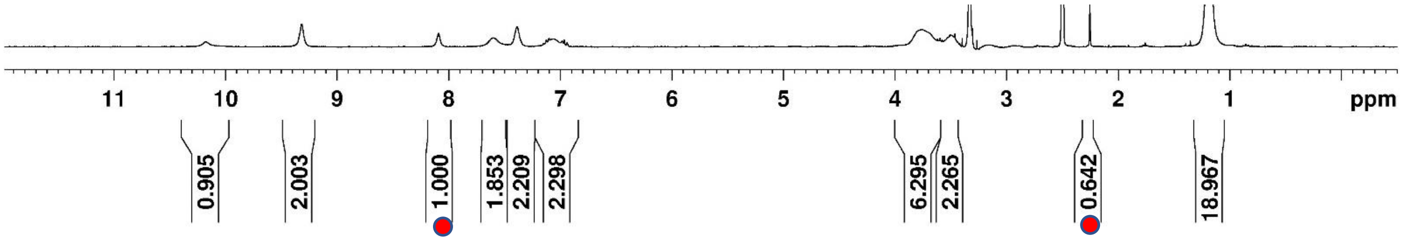

^{1}H-NMR of **TAPM-4Den** after adsorbing *m*-xylene in DMSO-D$_6$

(G) adsorbing *p*-xylene:

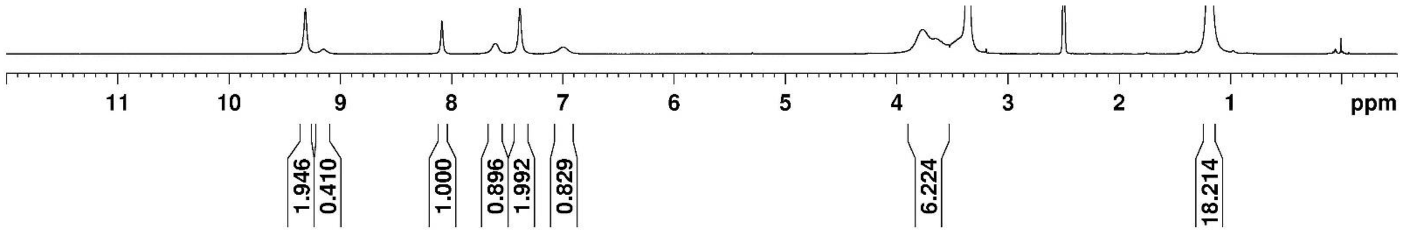

^{1}H-NMR of **TAPM-8Den** DMSO-D$_6$

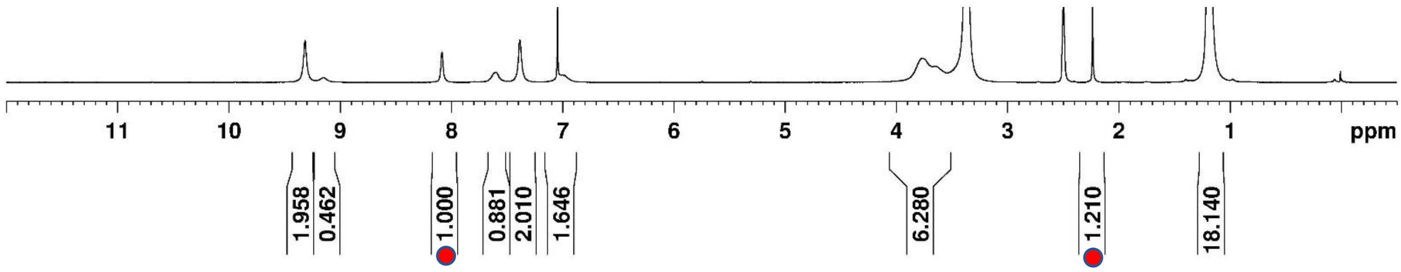

^{1}H-NMR of **TAPM-8Den** after adsorbing *p*-xylene in DMSO-D$_6$

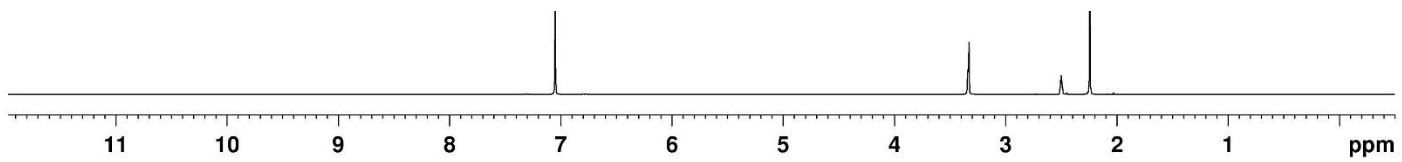

^{1}H-NMR of *p*-xylene in DMSO-D$_6$

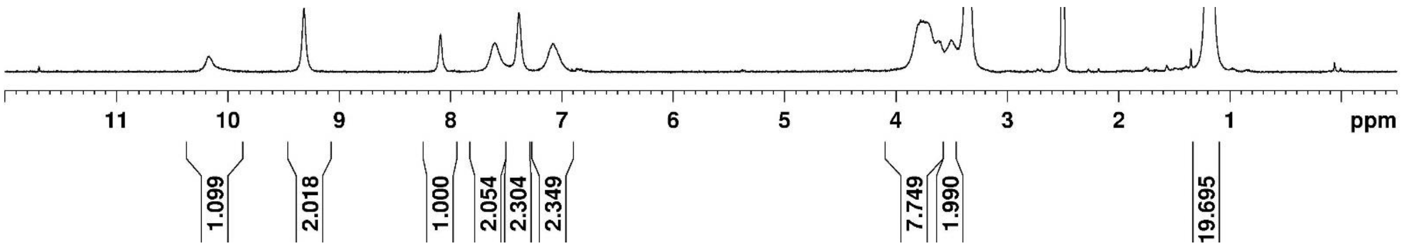

^{1}H-NMR of **TAPM-4Den** in DMSO-D$_6$

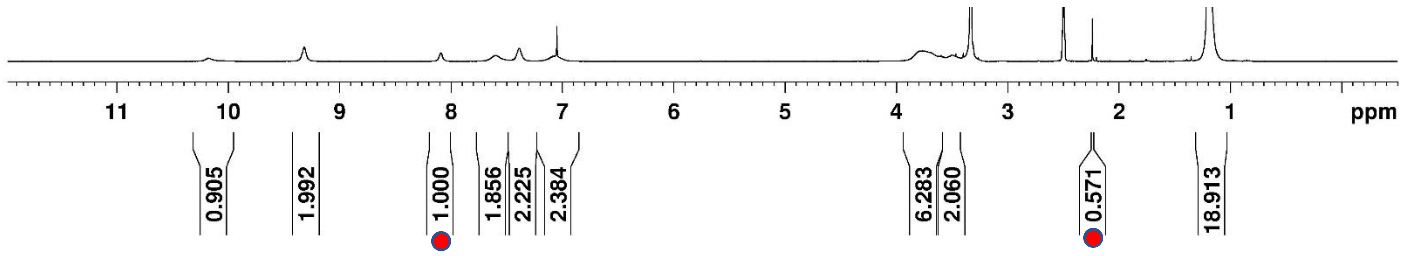

^{1}H-NMR of **TAPM-4Den** after adsorbing *p*-xylene in DMSO-D$_6$

Figure S6. The ^{1}H-NMR and ^{13}C-NMR spectra of dendrimers **TAPM-8Den** and **TAPM-4Den.**

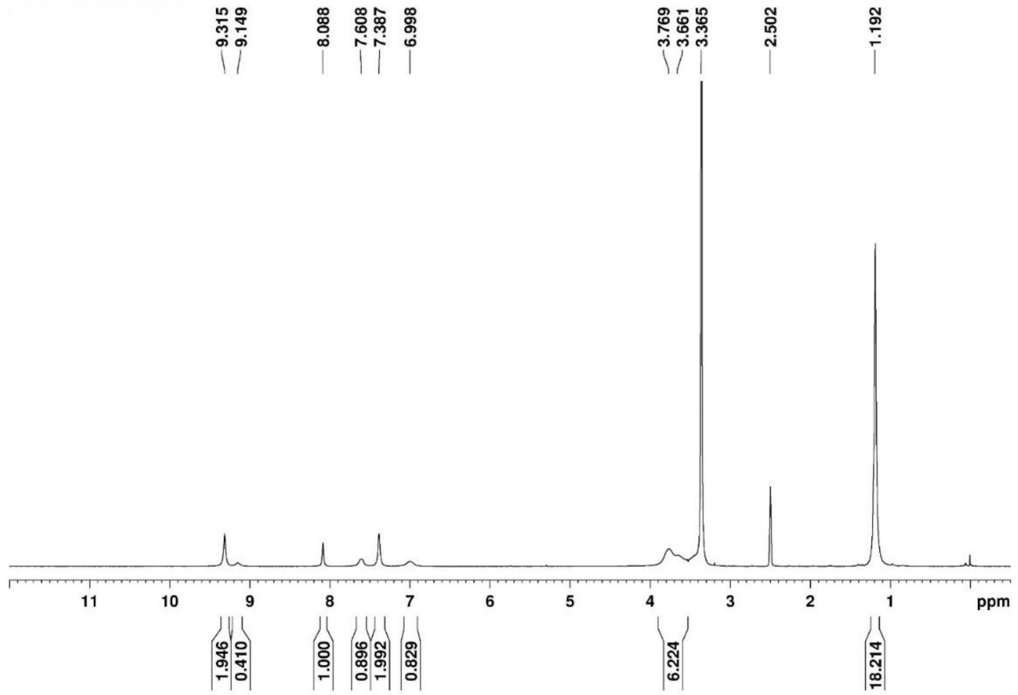

^{1}H-NMR spectrum of **TAPM-8Den**

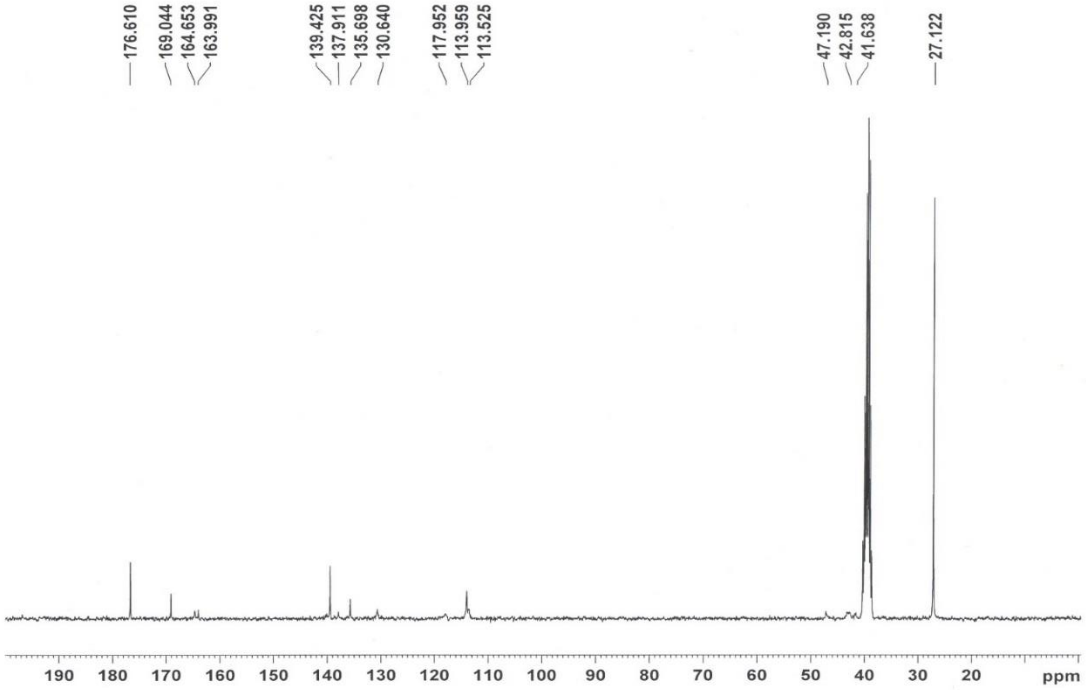

^{13}C-NMR spectrum of **TAPM-8Den**

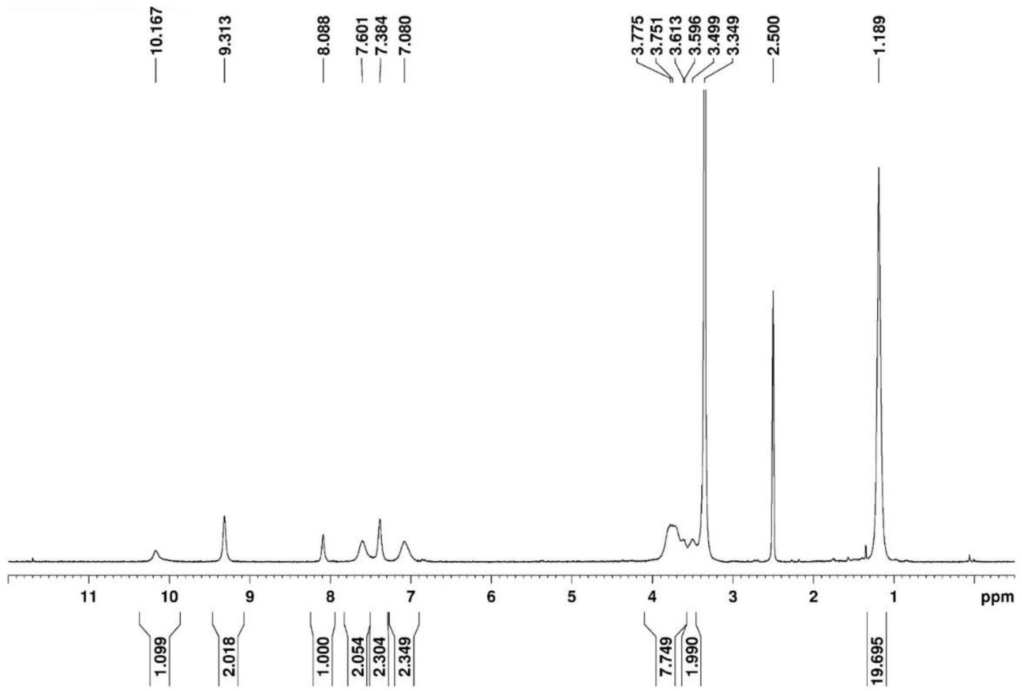

^{1}H-NMR spectrum of **TAPM-**4**Den**

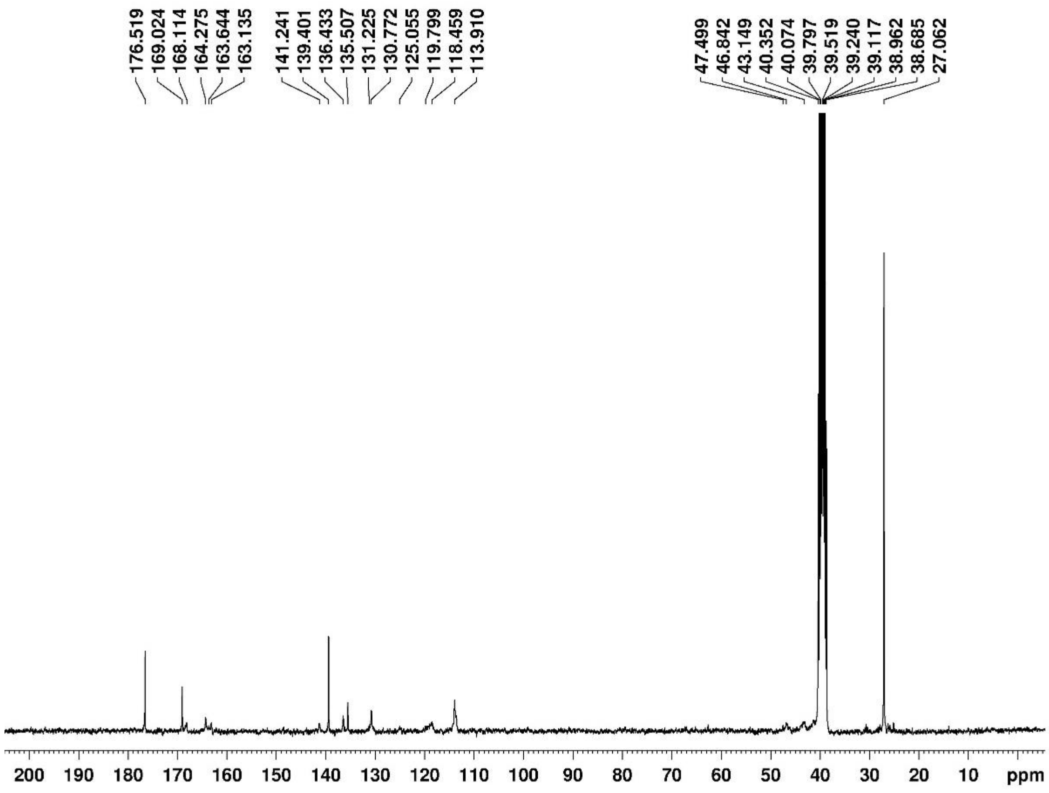

^{13}C-NMR spectrum of **TAPM-4Den**

Section S1. Estimation of isosteric heats of gas adsorption.

A virial-type Equation (S1) comprising the temperature independent parameters *ai* and *bi* was employed to calculate the enthalpies of adsorption for CO_2 (at 273 and 298 K).

$$\ln P = \ln N + 1/T \sum_{i=0}^{m} a_i N^i + \sum_{i=0}^{n} b_i N^i \quad \text{(S1)}$$

P: pressure, *N*: the amount adsorbed (or uptake), *T*: temperature, a_i and b_i: virial coefficients, and *m*, *n*: the number of coefficients required to adequately describe the isotherms (*m* and *n* were gradually increased until the contribution of the extra added *a* and *b* coefficients was deemed statistically insignificant towards the overall fitting, and the average value of the squared deviations from the experimental values was minimized). The values of the virial coefficients (a_0 to a_m) were then used to calculate the enthalpies heats of adsorption using the following expression.

$$Q_{st} = -R \sum_{i=0}^{m} a_i N^i \quad \text{(S2)}$$

Qst is the coverage-dependent isosteric heat of adsorption and *R* is the universal gas constant. The heat of CO_2 sorption for (S1) and (S2) in this manuscript is determined by using the excess sorption data in the pressure range from 0–1 atm (273 and 298 K), which is fitted by the virial-equation very well.
